# Knowledge, attitudes and practices regarding depression among primary health care providers in Fako division, Cameroon

**DOI:** 10.1186/s12888-018-1653-7

**Published:** 2018-03-13

**Authors:** Isabelle Dibu Mulango, Julius Atashili, Bradley N. Gaynes, Tsi Njim

**Affiliations:** 1 District Hospital Kumba, Kumba, South West Region Cameroon; 2 Medicine Programme, Faculty of Health Sciences, University of Buea, Buea, Cameroon; 3 Department of Psychiatry, University of North Carolina School of Medicine, Chapel Hill, North Carolina USA; 4 Health and Human Development Research Group, Douala, Littoral Region Cameroon; 5 Nuffield Department of Health, Centre for Global Health and Tropical Medicine, University of Oxford, Oxfordshire, UK

**Keywords:** Primary health care providers, Depression, Knowledge, Attitudes, Practices, Fako, Cameroon

## Abstract

**Background:**

Mental health and mental illness are often overlooked in the management of patients in our health services. Depression is a common mental disorder worldwide. Recognising and managing mental illnesses such as depression by primary health care providers (PHCPs) is crucial. This study describes the knowledge, attitudes and practices (KAP) of PHCPs regarding depression in Fako Division.

**Methods:**

A cross-sectional study was conducted among PHCPs (general practitioners, nurses, pharmacy attendants and social workers) in public-owned health facilities in the four health districts in Fako Division. Participants were selected by a consecutive convenience sampling. A structured questionnaire including the Depression Attitude Questionnaire (DAQ) was used to collect information about their socio-demographic characteristics, professional qualifications and KAP about depression.

**Results:**

The survey had a response rate of 56.7%. Most of the 226 participants (92.9%) were aware that depression needs medical intervention. Only 1.8% knew a standard tool used to diagnose depression. Two-thirds agreed that majority of the cases of depression encountered originate from recent misfortune. About 66% felt uncomfortable working with depressed patients. Also, 45.1% of PHCPs did not know if psychotropic drugs were available at pharmacies within their health area. Very few (15.2%) reported to have prescribed psychotropic drugs. Less than half (49.1%) of the participants had prior formal training in mental health.

**Conclusion:**

PHCPs in Fako Division tend to have limited knowledge and poor attitudes regarding depression. Practices towards diagnosis and management of depression tend to be inadequate. There is an urgent need to train PHCPs in mental health in general and depression diagnosis and management in particular.

**Electronic supplementary material:**

The online version of this article (10.1186/s12888-018-1653-7) contains supplementary material, which is available to authorized users.

## Background

Health is defined by the World Health Organization (WHO) as “a state of complete physical, mental and social well-being and not merely the absence of disease or infirmity” [1]. Mental health is an inherent component of health.

However, screening and treatment for depression and other mental health diseases in general are limited in sub-Saharan Africa. Research concerning routine screening of depression indicates benefits were greater among the general adult population, pregnant and postpartum women than in older adults, nonetheless accurate screening of all populations and provision of treatment were recommended [2]. Few studies have been done on the prevalence of mental disorders and associated factors [3–5]. In Cameroon, neuropsychiatric disorders are estimated to contribute to 6.1% of the global burden of disease. There are no official data on the prevalence of depression in the general population [6]. Furthermore, health care providers’ attitudes towards mental disorders have not been adequately assessed even though adequate knowledge on mental health by primary health care providers (PHCPs) is vital for the provision and integration of mental health services [7]. This is important as PHCPs are usually the first to encounter patients.

In Zambia, a recent study showed that among PHCPs, there was great stigma and discrimination towards mental illness [8]. Another study in Tanzania showed that the number of depressed persons is reported to have increased. The authors concluded on the need for PHCPs to undergo more training to ameliorate the management of depression [9].

While mortality is still mostly the result of infectious diseases and malnutrition, the morbidity associated to mental illnesses receives minimal attention from most governments. As a result, mental health services are poorly developed [3, 10]. It is projected that depression will cause the highest burden of disease by 2020 [11]. Mental illnesses-associated morbidity thereby requires that public policy makers and PHCPs change their attitudes towards mental and neurological disorders [3, 12].

In Cameroon, to the best of our knowledge, the knowledge, attitudes and practices (KAP) among PHCPs towards depression has not been assessed. We conducted this study to describe the KAP regarding depression among PHCPs to address these information gaps. As the range of mental disorders is vast, depression was used as representative of mental disorders. Information on current perceptions, diagnosis and management for depression, and mental health services may have an impact on the overall management of depression in a way best to suite our context and potentially improve mental health care in Cameroon.

## Methods

### Study design and setting

Fako Division is one of the six divisions of the South West Region of Cameroon. Fako Division has a population of about 534,854 people and an area of 2093km^2^ [13]. Fako Division has four health districts namely: Buea, Limbe, Tiko and Muyuka health districts [14]. There are 37 functional public-owned health facilities out of 89 health facilities within the four health districts. These health facilities include health centers and hospitals which represent the peripheral and intermediate levels of the Cameroon health system classification. Data on the exact number of health personnel in these health facilities is unavailable. There are two mental health nurses within Fako Division, one mental health nurse in each of the regional hospitals in Buea and Limbe.

We conducted an observational cross-sectional study of all PHCPs within public-owned health facilities in the four health districts in Fako Division who consented voluntarily to participate. PHCPs were selected by a consecutive convenience sampling.

### Participants and sampling

The study population was made of PHCPs (all general practitioners, social workers, pharmacy attendants and nurses). This study population was deliberately chosen because they are expected to provide primary health care which includes early detection of mental illness. Eligible participants were PHCPs aged ≥21 years who provided informed written consent. PHCPs less than 21 years of age were excluded from the study.

Participants were enrolled at their work station between July and December 2013. Every eligible participant available was approached by the principal investigator. Participation was voluntary and participants who gave their consent were given questionnaires to fill and return within one week. Questionnaires were anonymous. The questionnaire was pilot tested prior to the survey proper. A total of 400 PHCPs agreed to participate in this study and 226 returned completely filled questionnaires.

### Data collection, variables and measurements

The study was carried out using a structured questionnaire (Additional file 1) which was self-administered. The questionnaire was divided into four main sections. Section one which collected information on the key socio-demographic variables of the PHCPs (age, gender, marital status, profession, and duration of practice). Section two had 15 questions to assess the knowledge of PHCPs about depression. Section four had twelve items on the practice about depression and prior training in mental health. Knowledge and practice questions were selected from questions found in other studies [8, 15–18] and the questions were modified or rephrased for better understanding.

Section three used the DAQ to assess the attitudes of the PHCPs to depression. The DAQ was originally devised for use in the United Kingdom and it has been used successfully by various health professionals in other developed countries [19–21] as well as in African settings [9, 22]. The initial DAQ was a self-reported measure comprising of 20 items spanning 100 mm on a visual analogue scale ranging from “strongly agree” to “disagree” [23]. Sections two, three and four had questions were in ‘Agree/yes’, ‘Disagree/no’ or ‘Neutral/I don’t know’ format. This format was used in among PHCPs in Tanzania and GPs in Nigeria [9, 22].

### Data management and statistical analysis

Data from the questionnaires were entered into an Epi-Info version 7.0 database and analyzed using Epi-Info Version 7.0, Simple Interactive Statistical Analysis (SISA) and Microsoft Excel. Participants’ characteristics were described using absolute and relative frequencies of various responses for categorical variables and means and standard deviations were used for continuous variables such as age. The socio-demographic variables of PHCPs such as past training (on mental health and illness during their degree/certificate training) were compared to randomly selected KAP statements (amitriptyline is a psychotropic drug; depression reflects a characteristic response that is not amendable to change; you have prescribed a psychotropic drug to a patient with a depressive disorder). The association were assessed using Chi-square tests or Fisher’s exact tests as appropriate. Statistical significance was set at *p* < 0.05.

### Ethical approval

This research was approved by the Faculty of Health Sciences Institutional Reviews Board and the South West Regional Delegation of Public Health of Cameroon. Informed written consent was obtained from all participants. Participation was voluntary, and participants could withdraw at any time from the study.

The manuscript was written following the STROBE guidelines for reporting observational studies [24].

## Results

Of the 400 questionnaires distributed, 226 were returned (response rate = 56.7%). The characteristics of the 226 PHCPs who participated in the study are summarised in Table 1. Most (73.9%) of the participants were females. Almost half (41.6%) belonged to the age group 30–39 years. The mean age of study participants was 33.3 ± 7.6 years. PHCPs had an average duration of practice of 6.4 ± 6.4 years. A majority (61.1%) of them had worked for more than five years.

**Table 1:** Distribution of the study population according to socio-demographic variables

Variable	Category	Frequency	Proportion (%)
Gender	Male Female	59 167	16.1 73.6
Marital status	Single Married Widow Divorced	196 96 96 7	52.7 42.5 3.1 1.8
Age (years)	21–29 30–39 40–49 50–59	83 94 39 10	36.7 41.6 17.2 4.5
Duration of practice (years)	≤ 1 2–5 6–9 10–14 15–19 20–24 25–29 ≥30	47 91 34 31 8 8 5 2	20.8 40.3 15.0 13.7 3.5 3.5 2.2 1.0
Profession	Nurses GPs Social Workers Pharmacy attendants	184 25 7 10	81.4 11.1 3.1 4.4

A vast majority (81.4%, *n* = 184) of the respondents were nurses with 11.1% (*n* = 25) being general practitioners (GPs), 3.1% (*n* = 7) social workers and pharmacy attendants (4.4%, *n* = 10). Nurses include: state registered nurses and nurse assistants.

### Knowledge towards etiology of depression

Table 2 details the KAP towards depression. Majority (85.4%) disagreed that depression is caused by witchcraft, charms or evil spirits. 92.9% reported that depression can lead to suicide or suicide attempts.

**Table 2:** Responses to knowledge items of 226 PHCPs who responded to a survey on the Knowledge, Attitudes and Practices regarding depression in Fako Division, Cameroon

No.	Statement	Agree n (%)	Disagree n (%)	Don’t know^a^ n (%)	Total n (%)
1	Have you ever heard about depression?	226 (100)	0 (0)	0(0)	226 (100)
2	Do you consider depression as a health problem?	210 (92.9)	11 (5)	5 (2.1)	226 (100)
3	Depression affects people of a particular age group.	60 (26.5)	152 (67.3)	14 (6.2)	226 (100)
4	Depression is caused by witchcraft, charms or evil spirits.	14 (6.2)	193 (84.4)	19 (8.4)	226 (100)
5	Patients with depression can break down at any time.	203 (89.8)	12 (5.3)	11 (4.9)	226 (100)
6	Patients with depression are dangerous to themselves and others.	167 (73.9)	48 (21.2)	11 (4.9)	226 (100)
7	Depression can lead to suicide and suicide attempts.	210 (92.9)	12 (5.3)	1 (1.9)	226 (100)
8	Depression can be treated with pharmacological methods and psychotherapy.	194 (85.9)	18 (8.0)	14 (6.1)	226 (100)
9	Depression is best managed by traditional doctors/healers.	5 (2.2)	206 (91.2)	15 (6.6)	226 (100)
10	Depression responds better to traditional remedies than orthodox treatment most of the time.	8 (3.5)	181 (80.1)	37 (16.4)	226 (100)
11	Amitriptyline is an anti-depressant drug.	91 (40.3)	15 (6.6)	120 (53.1)	226 (100)
12	Methotrexate is an anti-depressant drug.	35 (15.5)	58 (25.7)	133 (58.9)	226 (100)
13	Fluoxetine is an anti-depressant drug.	64 (28.3)	23 (10.2)	139 (61.5)	226 (100)
14	Carbamazepine is an anti-depressant drug.	91 (40.3)	23 (10.2)	112 (49.5)	226 (100)
15	Do you know of a tool used to classify depressed patients? If response is yes,please specify the tool.	4 (1.8)	222 (98.2)	NA	226 (100)

^a^or Neutral; NA: Not applicable

### Knowledge about the treatment and classification of depression

Majority (85.8%) accepted that depression can be treated with pharmacological methods and psychotherapy. Only 28.3% agreed that fluoxetine is an anti-depressant drug.

### Attitudes towards the prevalence and origin of depression

Attitudes towards depression for each item are listed in Table 3. More than a third (45.1%) reported to have seen an increase in the number of depressed patients presenting with depressive symptoms during the last five years. More than 60% agreed that the majority of depressed cases they see originated from recent misfortune and perceived that depressed patients are more likely to have experienced deprivation in early life than other people. However, few (31.9%) felt that patients with depression are discriminated by and avoided by the general public.

**Table 3:** Participants’ responses to Depression Attitude Questionnaire

No.	Statement	Agree n (%)	Disagree n (%)	Don’t know^a^ n (%)	Total n (%)
1	During the last 5 years I have seen an increase in the number of patients presenting with depressive symptoms	102 (45.1)	93 (41.2)	31 (13.7)	226 (100)
2	The majority of depression cases I see originated from recent misfortune	146 (64.6)	51 (22.6)	29 (12.8)	226 (100)
3	Biochemical abnormality is at the basis of more severe depression	80 (35.4)	66 (29.2)	80 (35.4)	226 (100)
4	It is possible to distinguish two groups of depression, one psychological in origin and the other caused by biochemical mechanisms.	139 (61.5)	40 (17.7)	47 (20.8)	226 (100)
5	Depressed patients are more likely to have experienced deprivation in early life than other people.	134 (59.6)	59 (26.2)	32 (14.2)	226 (100)
6	Becoming depressed is a way that people with poor stamina deal with life difficulties	116 (51.6)	73 (32.4)	36 (16.0)	226 (100)
7	Becoming depressed is a natural part of becoming old	60 (26.6)	155 (68.6)	11 (4.8)	226 (100)
8	Are patients with depression discriminated by the general public and avoided?	72 (31.9)	128 (56.6)	26 (11.5)	26 (11.5)
9	Difficult to differentiate unhappiness or a clinical depressive disorder that needs treatment	90 (39.8)	96 (42.5)	40 (17.7)	226 (100)
10	Depression reflects a characteristic response which is not amendable to change	35 (15.5)	149 (65.9)	23 (10)	226 (100)
11	Antidepressants usually produce a satisfactory result in the treatment of depressed patients in general practice	139 (61.5)	44 (19.5)	43 (19.0)	226 (100)
12	Most depressive disorders improve without medication	152 (67.3)	50 (22.6)	24 (10.6)	226 (100)
13	The primary health care worker could be a useful person to support depressed patients	197 (87.2)	17 (7.5)	12 (5.3)	226 (100)
14	There is little to be offered those depressed patients who do not respond to what primary health care workers do	64 (28.4)	122 (54.2	39 (17.3)	226 (100)
15	If depressed patients need antidepressants, they are better off with psychiatrists than with primary health care workers	162 (71.7)	30 (13.3)	34 (15.0)	226 (100)
16	Psychotherapy for depressed patients should be left to a specialist	154 (64.4)	58 (25.8)	13 (5.7)	226 (100)
17	If psychotherapy were freely available, this would be more beneficial than antidepressants for most depressed patients	162 (71.7)	30 (13.3)	34 (15.0)	226 (100)
18	I feel comfortable dealing with depressed patients	60 (26.6)	149 (65.9)	17 (7.5)	226 (100)
19	Working with depressed patients is heavy going, tedious or difficult	168 (74.3)	45 (19.9)	13 (5.8)	226 (100)
20	It is rewarding to spend time looking after depressed patients	148 (65.5)	49 (21.7)	29 (12.8)	226 (100)

^a^= or Neutral

### Attitudes about the diagnosis and prognosis of depression

Less than half (42.5%) of the respondents disagreed that it is difficult to differentiate unhappiness from a clinical depressive disorder that needs treatment. More than half (61.5%) perceived that antidepressants usually produce a satisfactory result in treatment of depressed patients.

### Attitudes regarding treatment of depression

Nearly two thirds (67.3%) believed that most depressive disorders improve without medication. A vast majority (82.7%) endorsed the statement that PHCPs could be a useful person to support depressed patients. More than 60% of PHCPs agreed that if depressed patients need antidepressants or psychotherapy they are better off with psychiatrists.

### Working with depressed patients

Only 26.5% of PHCPs felt comfortable dealing with depressed patients and a majority (74.3%) agreed that working with depressed patients is “heavy going”, tedious or difficult. However, nearly two thirds endorsed the statement that it is rewarding to spend time looking after depressed patients.

### Practices towards recognition and management of depression

Very few (12%) of PHCPs were routinely screening patients for depression, while 16.4% never screened patients for depression. More than a third (43.8%) of the participants agreed that time constraints is a limiting factor to managing depressed patients.

Majority (78.4%) reported that they would send patients with depression for counseling or medical management, while 21.6% of the sample would send depressed patients home, for prayers or to traditional healers.

### Training in mental health and illness

**Fig. 1:**
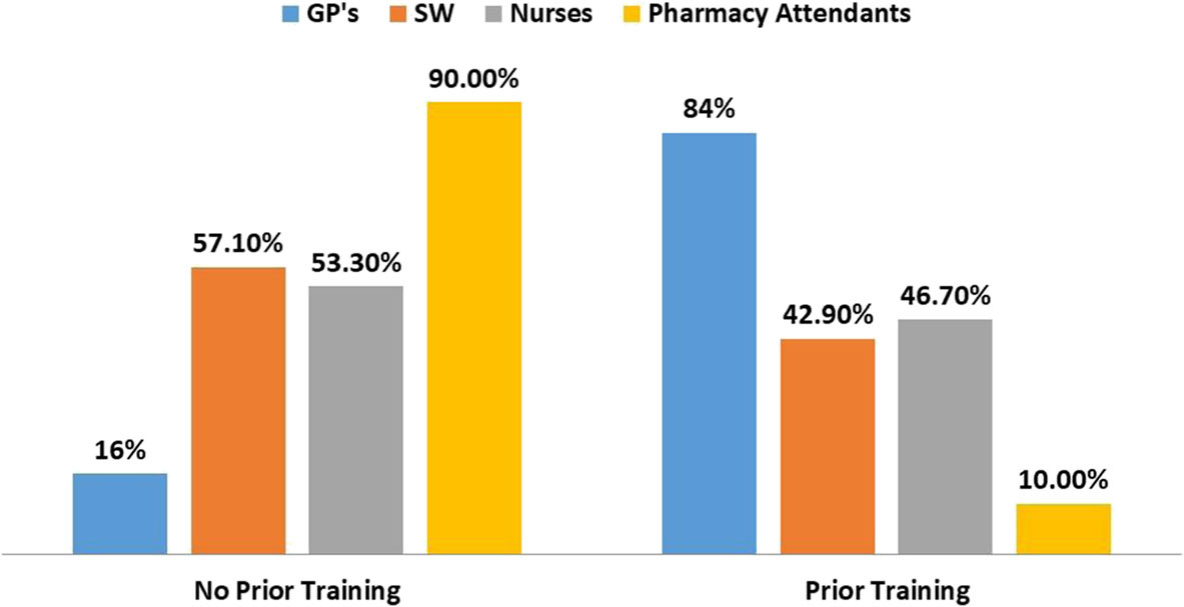
PHCPs training on Mental Health and Illnesses during undergraduate studies in Fako Division, Cameroon

**Fig. 2:**
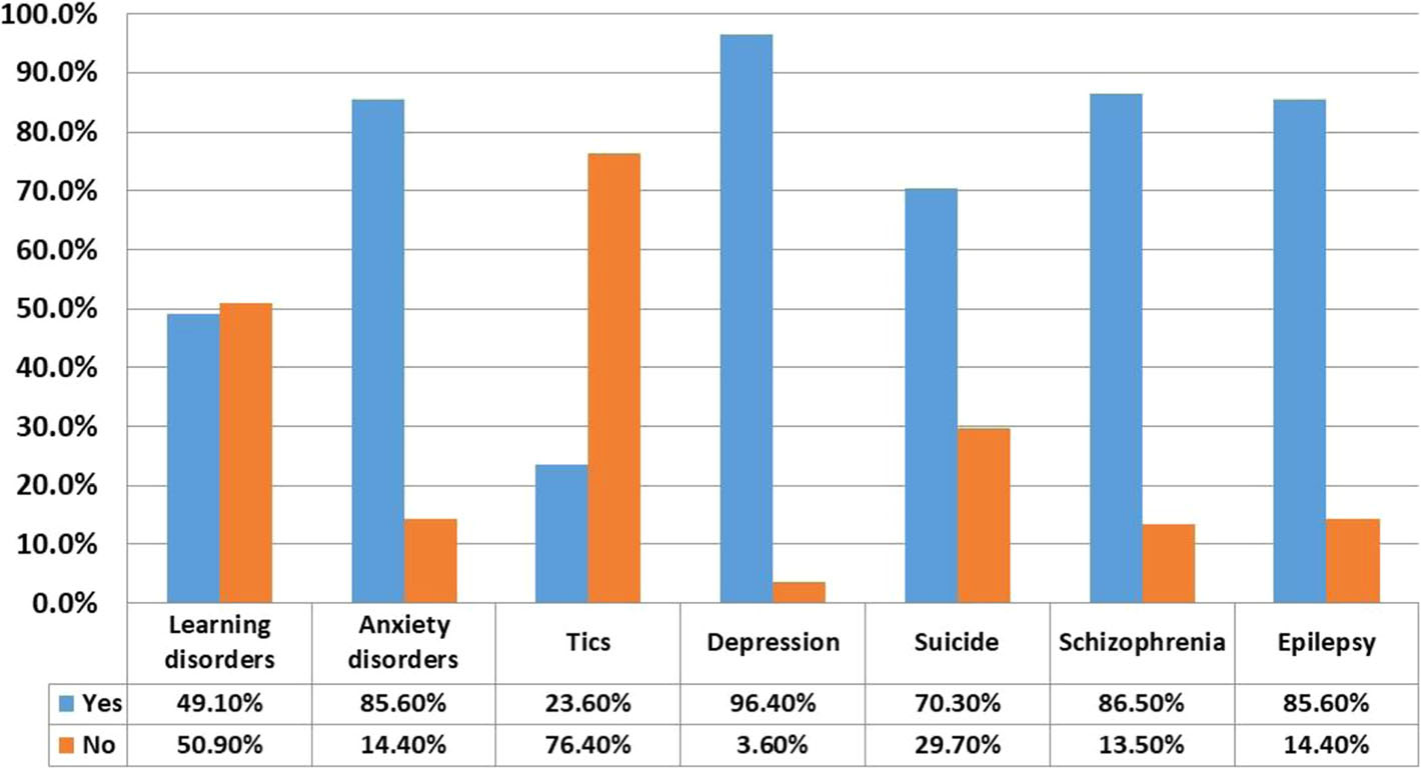
Distribution of prior mental health training and mental disorders covered by PHCPs in Fako Division, Cameroon

Less than half (49.1%) of the sample had prior training in mental health during their undergraduate studies: 84% of GPs and 46.7% of all nurses (Fig. 1). This training covered depression, anxiety disorders, schizophrenia and epilepsy (Fig. 2). However, only 1.8% knew of a standard tool used in the classification of depression such as the Diagnostic Statistical Manual Fourth Edition (DSM-IV), Neurobehavioral functioning inventory Yale tool (NFI), Hospital anxiety and depression scale (HAD).

All the PHCPs with prior undergraduate training agreed that the training on mental health and disorders was less as compared to the time spent on training in infectious diseases and chronic illnesses. Only 0.9% (*n* = 2) of the respondents went for further training on mental health after undergraduate studies. One general practitioner had a 6 months training on mental health and one mental health nurse had 1-year training on mental health.

### Availability and prescription of psychotropic drugs

Few (6.6%) agreed that psychotropic drugs were always available at pharmacies within their health area. Furthermore, 15.1% agreed to have prescribed psychotropic drugs such as amitriptyline, fluoxetine, haloperidol and risperidone. GPs prescribed psychotropic drugs more than any other category of health professionals.

### Associations of selected KAP items with some socio-demographic variables

PHCPs who had prior training during their undergraduate training were significantly more likely to know that amitriptyline is a psychotropic drug (OR = 0.34, 95% CI [0.19–0.6], *p* = 0.001). In addition, these PHCPs were more likely to agree that depression reflects a characteristic response that is amendable to change (OR = 2.0, 95%CI [0.95–4.23], *p*-value = 0.03). Also, these PHCPs were significantly more likely to prescribe psychotropic drugs (OR = 3.45, 95% CI [1.52–7.81], *p* = 0.003).

## Discussion

Participants’ knowledge on depression was generally low in our study. Attitudes regarding depression reported negative responses and PHCPs had limited training on mental disorders. Depression is a significant public health problem worldwide [11]. PHCPs play a major role in the diagnosis and management of depression.

In this study, PHCPs have good knowledge on the causation of depression in general. There were some misconceptions on depression, as a vast majority agreed that depressed patients can breakdown at any time [15].

Regarding the knowledge on management of depression, the large majority knew the effectiveness of pharmacological methods and psychotherapy. However, few PHCPs were knowledgeable about a standard tool used in the screening of depression. Recent evidence suggest PHCPs should use a standardized screening tool for depression to reduce underdiagnosis and to determine the diagnosis of depression with DSM-V criteria to avoid overuse and unneeded detrimental effects of pharmacotherapy [2]. The underdiagnosis of depression has been associated with increased morbidity in other studies [9, 16, 22]. Our data are in contrast with those of Seehusen et al. in the United States where a higher proportion (30.6%) of family physicians used a validated screening tool for postpartum depression [25]. Moreover, the use of validated tools has proven to be important in screening for depression [21, 26, 27]. Furthermore, only few of the respondents knew that amitriptyline, an anti-depressant is a psychotropic drug and similar results were observed in Nigeria [28].

Less than half of the respondents reported an increase in the number of depressed patients, and this has been observed in prior studies among non-psychiatric doctors in Japan and health workers in Tanzania [9, 29]. Although the prevalence of depression in the general population of Cameroon is not known [6], PHCPs recognize the burden of depression in primary health care. More data from future studies are needed to assess if the rate of depression is increasing in the general population. Recently, few studies have been carried out on the prevalence of depression in specific populations such as HIV/AIDS patients. A study conducted by Gaynes et al. in Bamenda, Cameroon among HIV-infected patients (*n* = 11) revealed a low prevalence of last month major depression (3%) among that specific sample [4]. Another study by L’akoa et al. in Yaounde, Cameroon revealed that the prevalence of depression (63%) among newly diagnosed HIV patients (*n* = 100) was higher [5]. Although both studies used the same cut-off score of ≥10 as a positive screen of depression with PHQ-9, the vast difference in the prevalence of major depression among HIV-infected patients in the above studies may be due to differences in sample size.

PHCPs had misconceptions on the risk factors and cause of depression. Most of the respondents believed that depression is caused by misfortune. Similar findings have been reported elsewhere [20, 30]. Discriminatory attitudes and stigma towards depression has been shown to limit depressed patients to seek help [7, 31]. The stigma associated with this condition could also explain the unwillingness of about half of the target population to participate in the study. A recent study in Canada and Cameroon revealed that persons from Cameroon had more stigmatizing attitudes towards mentally ill patients when compared to persons from Canada [17]. Variations in PHCPs and patients’ views on depression may strongly be influenced by social and cultural factors [15].

In our study, majority of PHCPs felt uncomfortable working with depressed patients. This is consistent with prior studies [22, 26]. One in three PHCPs reported to have difficulty differentiating unhappiness from a clinical depressive state that needs treatment. Our results are similar with a study among GPs in Brazil [32] but in contrast to GPs in France [20]. This can be accounted for by the fact that physicians in developed world have more training on mental illness.

Majority of the respondents preferred depressed patients to be managed by specialists. Similar findings were seen in other studies [9, 15, 29, 33]. PHCPs have limited skills to offer adequate management options hence prefer the management of depression by mental health professionals.

Regarding practices, only few PHCPs agreed to always screen patients for depression. PHCPs identified time constraints, limited skills and training as limitations in the diagnosis and management of depression as earlier documented [11, 15]. The workforce in the mental health sector in Cameroon is severely limited and cannot meet the needs to care for mentally ill patients [6]. In the United Kingdom, a study done by Haddad et al. reported that if nurses in general practice were provided adequate training in mental health, they could provide better mental health services [23], supporting the findings of other studies [27, 28, 34, 35].

The two main mental health treatment centers in the Cameroon: Jamot Hospital Yaounde and Laquintinie Hospital Douala are found in the Center and Littoral Regions respectively [14]. Hence referral from primary care to specialized mental health services is inadequate [15, 36].

About a third of the PHCPs agreed that psychotropic drugs were available at pharmacies within their health area. Studies by Olugbile et al. report that only a fifth of GPs had psychotropic drugs available in their drug stocks [18]. This can be linked to the fact that psychotropic drugs are limited in primary health care.

The study had the following limitations. This study used a non-random sampling method and was done among PHCPs working in public-owned health facilities in the four health districts in Fako Division in the South West Region of Cameroon, these results may not be a representation of the rest of the country. The DAQ was first used in United Kingdom. Although it has been widely used with GPs and health workers success in African settings, the scale may not have been transferable to a different country or culture. We did not evaluate the validation (general test score characteristics, internal reliability, factor structure) of the DAQ nor it’s psychometric properties. In addition, there have been questions on the psychometric inadequacy of the DAQ in relation to its differing subscales and modest internal consistency [37]. However, using the DAQ provided us with the opportunity to compare findings in studies in African settings and elsewhere which implemented the same tool. Thus, there may be a need to carry out a larger survey among PHCPs in Cameroon using a revised version of the DAQ [30] assessing psychometric properties and accounting for some of its psychometric inadequacies.

The survey’s low response rate can be accounted by participants unwillingness to participate due to the stigma associated with depression. Also, there was a problem of recall bias as majority of those who had training in mental health could not correctly estimate the duration of training or type of mental illness studied. These limitations should be considered when interpreting the results.

Notwithstanding these limitations, this paper does present new data on the KAP among primary health care providers regarding depression.

### Implications

Educational intervention programs in areas of mental health to improve the knowledge, attitudes and practices of PHCPs towards depression is paramount. If this is implemented, PHCPs will be more confident in dealing with depressed cases. Previous studies have shown that continued medical education is linked to better diagnosis and management of depressed patients.

## Conclusions

PHCPs have insufficient KAP about the management of depression. There was paucity in depression management options. PHCPs generally had negative views regarding depression. PHCPs felt uncomfortable and perceived difficulty when working with depressed patients. This study revealed that in practice, PHCPs do not or rarely screen or assess patients for depression, which is a very common mental disorder encountered within primary health care. PHCPs had limited training in mental health. We recommend that, the government through the Ministry of Public Health should draft and implement a national mental health policy. In addition, the resources allocated to mental health should be increased and the creation of more mental health services. We also recommend the provision of schools and training programs in the area of mental health, to increase the number of mental health professionals. Furthermore, the creation of awareness education programmes on mental illnesses.

## Additional file

Additional file 1: Mental Health Questionnaire. Title of data: Questions used during the study “Knowledge, Attitudes and Practices regarding Depression among Primary Health Care Providers in Fako Division, Cameroon”. Description of data: The study was carried out using a 52 items structured questionnaire which was self-administered from July 2013 to December 2013. The questionnaire was divided into four main sections. Section one which collected information on the key socio-demographic variables of the PHCPs. Section two had 15 items to assess the knowledge of PHCPs about depression. Section four had 12 items on the practice about depression and prior training in mental health. The knowledge and practice items were selected from questions found in other studies [8, 15–18] and the questions were modified or rephrased for better understanding. Section three used the DAQ to assess the attitudes of the PHCPs to depression. The DAQ was originally devised for use in the United Kingdom and it has been used successfully by various health professionals in other developed countries [19–21] as well as in African settings [9, 22]. (DOCX 23 kb)

## Abbreviations

AIDS: Acquired immune deficiency syndrome
DAQ: Depression attitude questionnaire
DSM-IV: Diagnostic Statistical Manual Fourth Edition (DSM-IV) DSM-V Diagnostic Statistical Manual Fourth Edition (DSM-V)
GP: General practitioner
HAD: Hospital anxiety and depression scale (HAD)
HIV: Human immuno-deficiency virus
KAP: Knowledge, attitudes and practices
NFI: Neurobehavioral functioning inventory
PHCP: Primary health care providers
SISA: Simple Interactive Statistical Analysis
WHO: World Health Organization
