# Supplementary material for: Knowledge, attitudes and practices regarding depression among primary health care providers in Fako division, Cameroon
Source: BMC Psychiatry. 2018 Mar 13;18:66. doi: 10.1186/s12888-018-1653-7 (PMC5850974; doi:10.1186/s12888-018-1653-7)
Supplement: Supplementary file 1 — Mental Health Questionnaire. Title of data: Questions used during the study “Knowledge, Attitudes and Practices regarding Depression among Primary Health Care Providers in Fako Division, Cameroon”. Description of data: The study was carried out using a 52 items structured questionnaire which was self-administered from July 2013 to December 2013. The questionnaire was divided into four main sections. Section one which collected information on the key socio-demographic variables of the PHCPs. Section two had 15 items to assess the knowledge of PHCPs about depression. Section four had 12 items on the practice about depression and prior training in mental health. The knowledge and practice items were selected from questions found in other studies [8, 15–18] and the questions were modified or rephrased for better understanding. Section three used the DAQ to assess the attitudes of the PHCPs to depression. The DAQ was originally devised for use in the United Kingdom and it has been used successfully by various health professionals in other developed countries [19–21] as well as in African settings [9, 22]. (DOCX 23 kb) [file 12888_2018_1653_MOESM1_ESM.docx]

**Manuscript title: Knowledge, Attitudes and Practices regarding Depression among Primary Health Care Providers in Fako Division, Cameroon**.

**Study Questionnaire**

Health district____________ Health area____________

Health facility____________

Participant number_________

Please circle your answer.

**SECTION 1: Demographics**

(1) What is your age? ________________ (years)

(2) What is your sex?

(a) Female

(b) Male

3) What is your Marital status (please check one).

(a) Single

(b) Married

(c) Widowed

(d) Divorced

(4) What is your Professional rank: a) General practitioner (GP) b) social worker

c) State registered nurse c) Nursing assistant d) Pharmacy attendants

(5) Duration of practice (in years) ____________

**SECTION 2: Knowledge on diagnosis, management of depression.**

| **Question Number** | **QUESTIONS** | **Yes (Agree)** | **No (Disagree)** | **Don’t Know** |
| --- | --- | --- | --- | --- |
| 1 | Have you ever heard about depression? |  |  |  |
| 2 | Do you consider depression as a health problem? |  |  |  |
| 3 | Depression affects people of a particular age group |  |  |  |
| 4 | Depression is caused by witchcraft, charms, evil spirits |  |  |  |
| 5 | Patients with depression can breakdown at anytime |  |  |  |
| 6 | Patients with depression are dangerous to themselves and others |  |  |  |
| 7 | Depression can lead to suicide or suicide attempts |  |  |  |
| 8 | Depression can be treated with pharmacological methods and psychotherapy |  |  |  |
| 9 | Depression is best managed by traditional doctors/healers |  |  |  |
| 10 | Depression respond better to traditional remedies than orthodox treatment most of the time |  |  |  |
| 11 | Amitriptyline is an anti-depressant drug |  |  |  |
| 12 | Methotrexate is an anti-depressant drug |  |  |  |
| 13 | Fluoxetine is an anti-depressant drug |  |  |  |
| 14 | Carbamazepine is an anti-depressant drug |  |  |  |

(15) Do you know of any mental health facility?

(a) Yes (b) No

If response is (a), please specify which mental health facility and include location (list all you know) ________________________

**SECTION 3: ATTITUDES TOWARDS IDENTIFICATION, DIAGNOSING AND MANAGEMENT OF DEPRESSION**

| **Question** | **Depression attitude questionnaire (DAQ)** | **Disagree** | **Agree** | **Don’t Know** |
| --- | --- | --- | --- | --- |
| 1 | During the last 5 years I have seen an increase in the number of patients presenting with depressive symptoms |  |  |  |
| 2 | The majority of depression cases I see originated from recent misfortune |  |  |  |
| 3 | Most depressive disorders improve without medication |  |  |  |
| 4 | Biochemical abnormality is at the basis of more severe depression |  |  |  |
| 5 | Difficult to differentiate unhappiness or a clinical depressive disorder that needs treatment |  |  |  |
| 6 | It is possible to distinguish two groups of depression, one psychological in origin and the other caused by biochemical mechanisms |  |  |  |
| 7 | Becoming depressed is a way that people with poor stamina deal with life difficulties |  |  |  |
| 8 | Depressed patients are more likely to have experienced deprivation in early life than other people |  |  |  |
| 9 | I feel comfortable dealing with depressed patients |  |  |  |
| 10 | Depression reflects a characteristic response which is not amendable to change |  |  |  |
| 11 | Becoming depressed is a natural part of becoming old |  |  |  |
| 12 | The primary health care worker could be a useful person to support depressed patients |  |  |  |
| 13 | Working with depressed patients is heavy going, tedious |  |  |  |
| 14 | There is little to be offered to depressed patients who do not respond to what primary health care workers do |  |  |  |
| 15 | It is rewarding to spend time looking after depressed patients |  |  |  |
| 16 | If depressed patients need antidepressants, they are better off with psychiatrists than with primary health care workers |  |  |  |
| 17 | Antidepressants usually produce a satisfactory result in the treatment of depressed patients in general practice |  |  |  |
| 18 | Psychotherapy for depressed patients should be left to a specialist |  |  |  |
| 19 | If psychotherapy were freely available, this would be more beneficial than antidepressants for most depressed patients |  |  |  |
| 20 | Patients with depression are discriminated in by the general public and avoided |  |  |  |

**SECTION 4: PRACTICES TOWARDS IDENTIFICATION, DIAGNOSIS AND MANAGEMENT OF DEPRESSION.**

1) Do you assess or screen patients who come to the health facility for depression?

(a) Never (b) Rarely (c) Sometimes (d) Always

2) Time constraints should not be a limiting factor to managing depressive patients.

(a) Agree (b) Disagree (c) I don’t know

3) If you identify a person with depression, you do not provide any management options. (Choose one)

(a) Agree (b) Disagree (c) I don’t know

4) You send patients with depressive disorders. (Choose one or more)

(a) Home (b) Prayers (c) Counseling (d) Traditional healers and practitioners (d) Offer medical management

5) Mental health services are provided within the health facility in which you work. (Choose one)

(a) Agree (b) Disagree (c) I don’t know

If your answer is “Agree” (a), please state the rank of the personnel(s) within the mental health service found in the health facility in which you work. (Choose one or more)

(a) Mental health nurse (b) State registered nurse (c) General practitioner (d) Psychiatrist (e) Psychologist

6) Did you have any formal training on mental health and illness during your degree/certificate training? (Choose one)

(a) Yes (b) No

If Yes (1), please specify if the training covered the following mental disorders

|  | Mental disorder | Yes | No |
| --- | --- | --- | --- |
| a) | Learning disorder |  |  |
| b) | Anxiety disorder |  |  |
| c) | Tics |  |  |
| d) | Depression |  |  |
| e) | Suicidal behaviors |  |  |
| f) | Schizophrenia |  |  |
| g) | Epilepsy |  |  |

Estimate in total how long the above training on mental health/illness last. ________________________hours.

7) Compare how much time this training in mental health was, compared to training on infectious diseases, cancer or diabetes. (Choose one)

(a) More (b) Equal (c) Less

8) Did you have any formal training on mental health and illness after your degree/certificate training? (Choose one)

(a) No (b) Yes

If Yes (b), please specify if the training covered the following mental disorders

|  | Mental disorder | Yes | No |
| --- | --- | --- | --- |
| a) | Learning disorder |  |  |
| b) | Anxiety disorder |  |  |
| c) | Tics |  |  |
| d) | Depression |  |  |
| e) | Suicidal behaviors |  |  |
| f) | Schizophrenia |  |  |
| g) | Epilepsy |  |  |

Estimate in total how long the above training on mental health/illness after your degree/certificate last. ________________________hours.

9) Compare how much time this training in mental health after your degree/certificate was, compared to training on infectious diseases, cancer or diabetes. (Choose one)

(a) More (b) Equal (c) Less

10) Primary health care providers need to have formal training on mental health and illness to improve on care to depressed patients. (a) No (b)Yes

11) Psychotropic drugs are available at pharmacies with our health area. (Choose one)

(a) Never (b) Sometimes (c) Always (d) I don’t know

12) Have you ever prescribed a psychotropic drug to a patient with depressive disorder? (Choose one)

(a) Never (b) Sometimes (c) Always (d) I don’t know

If response is (b) or (c), please specify which drug ________________________

Thank you for taking the time to complete this questionnaire.
